# Enhancement of Morphological Plasticity in Hippocampal Neurons by a Physically Modified Saline via Phosphatidylinositol-3 Kinase

**DOI:** 10.1371/journal.pone.0101883

**Published:** 2014-07-09

**Authors:** Avik Roy, Khushbu K. Modi, Saurabh Khasnavis, Supurna Ghosh, Richard Watson, Kalipada Pahan

**Affiliations:** 1 Department of Neurological Sciences, Rush University Medical Center, Chicago, IL, United States of America; 2 Revalesio Corporation, Tacoma, WA, United States of America; Centre national de la recherche scientifique, University of Bordeaux, France

## Abstract

Increase of the density of dendritic spines and enhancement of synaptic transmission through ionotropic glutamate receptors are important events, leading to synaptic plasticity and eventually hippocampus-dependent spatial learning and memory formation. Here we have undertaken an innovative approach to upregulate hippocampal plasticity. RNS60 is a 0.9% saline solution containing charge-stabilized nanobubbles that are generated by subjecting normal saline to Taylor-Couette-Poiseuille (TCP) flow under elevated oxygen pressure. RNS60, but not NS (normal saline), PNS60 (saline containing a comparable level of oxygen without the TCP modification), or RNS10.3 (TCP-modified normal saline without excess oxygen), stimulated morphological plasticity and synaptic transmission via NMDA- and AMPA-sensitive calcium influx in cultured mouse hippocampal neurons. Using mRNA-based targeted gene array, real-time PCR, immunoblot, and immunofluorescence analyses, we further demonstrate that RNS60 stimulated the expression of many plasticity-associated genes in cultured hippocampal neurons. Activation of type IA, but not type IB, phosphatidylinositol-3 (PI-3) kinase by RNS60 together with abrogation of RNS60-mediated upregulation of plasticity-related proteins (NR2A and GluR1) and increase in spine density, neuronal size, and calcium influx by LY294002, a specific inhibitor of PI-3 kinase, suggest that RNS60 upregulates hippocampal plasticity via activation of PI-3 kinase. Finally, in the 5XFAD transgenic model of Alzheimer’s disease (AD), RNS60 treatment upregulated expression of plasticity-related proteins PSD95 and NR2A and increased AMPA- and NMDA-dependent hippocampal calcium influx. These results describe a novel property of RNS60 in stimulating hippocampal plasticity, which may help AD and other dementias.

## Introduction

Alzheimer’s disease (AD) is the most common neurodegenerative disorder in the aged population. Impairments in memory and cognition, as well as extensive loss of hippocampal neurons [1] are the hallmarks of this disease. The death of hippocampal neurons is often associated with strong down-regulation of many functional genes [2] involved in ion conductance [3], [4], synapse formation [5], dendritic arborization [6], long term potentiation [7], [8], and long term depression [8], [9]. Impaired calcium influx through ionotropic glutamate receptors including NMDA and AMPA receptors is directly linked to the loss of hippocampal learning and memory [10]. Postmortem analysis of AD brains showed that expression of NMDA receptor subunits including NR1, NR2A, and NR2B was altered in susceptible brain regions including hippocampus [11]. Down-regulation of immediate early genes (IEGs) [12] including *arc*, *zif-268*, *homer-1*, *c-fos* and inhibition of synapse-associated genes [13], [14], [15] including *psd-95*, *synpo*, *adam-10* have also been reported in AD brain. In addition, oxidative [16] and nitrosylative [17], [18] damages in different hippocampal proteins also have been implicated in the loss of function and eventual death of hippocampal neurons. Many pharmacological compounds have been tested in the treatment of AD, including cholinesterase inhibitors and memantine, but most of them generate several side effects because of the poor metabolic activities of the elderly population.

RNS60 is physically modified saline that contains no active pharmaceutical ingredients. It is generated by subjecting normal saline to Taylor-Couette-Poiseuille (TCP) flow under elevated oxygen pressure [19], [20], [21]. Recently, we have demonstrated that RNS60 exerts anti-inflammatory effects in glial cells via suppression of nuclear factor kappa B (NF-κB) activation [19]. Here, we delineate that RNS60, stimulates calcium influx via NMDA- and AMPA-sensitive ionotropic glutamate receptors and increases the expression of many plasticity related genes in hippocampal neurons and thus may affect hippocampal plasticity. Furthermore, we demonstrate that RNS60 induced the activation of type IA PI-3 kinase and that RNS60 stimulated morphological plasticity and increased calcium influx in hippocampal neurons via PI-3 kinase activation. Finally, in the 5XFAD transgenic model of AD, RNS60 treatment upregulated plasticity-related molecules and increased hippocampal calcium influx. Our studies suggest that this physically-modified saline may be explored as a therapeutic agent in AD and other dementias.

## Materials and Methods

Animal maintaining and experiments were in accordance with National Institute of Health guidelines and were approved by the Institutional Animal Care and Use committee of the Rush University of Medical Center, Chicago, IL. Whenever needed, animals were anesthetized by ketamine/xylazine injectables.

### Reagents

Neurobasal medium and B27 supplement were purchased from Invitrogen (Carlsbad, CA). Other cell culture materials (Hank’s balanced salt solution, 0.05% trypsin and antibiotic-antimycotic) were purchased from Mediatech (Washington, DC). 5XFAD transgenic mice were purchased from Jackson Laboratory, genotyped and maintained in our animal care facility. Super array kit for analyzing mouse plasticity genes was purchased from SAbiosciences. Primary antibodies, their sources and concentrations used are listed in *Table 1*. Alexa-fluor antibodies used in immunostaining were obtained from Jackson ImmunoResearch and IR-dye-labeled reagents used for immunoblotting were from Li-Cor Biosciences. NMDA (cat #M3262), AMPA (cat #P8765) were purchased from Sigma-Aldrich. N20C hydrochloride (cat #2213) and naspm trihydrochloride (cat #2766) were purchased from TOCRIS.

**Table 1:** Antibodies, sources, applications, and dilutions used.

Antibody	Manufacturer	Catalogue#	Host	Application	Dilution/Amount
NR2A	Cell Signaling	4205	Rabbit	WB, ICC/IF	1∶500 (WB) 1∶100(IF)
GluR1	Cell Signaling	8850	Rabbit	WB, ICC/IF	1∶500 (WB) 1∶100(IF)
β-actin	Abcam	Ab6276	Mouse	WB	1∶6000
CREB	Cell Signaling	9197S	Rabbit	WB	1∶500
PSD95	Abcam	Ab2723	Mouse	WB, ICC/IF	1∶1000 (WB) 1∶100(IF)
PI3 Kinase p110α	Cell Signaling	4249S	Rabbit	WB	1∶1000
PI3 Kinase p110β	Santacruz Biotechnology	sc-7175	Rabbit	WB	1∶200
PI3 Kinase p110γ	Santacruz Biotechnology	sc-166365	Mouse	WB	1∶200

WB, Western blot; ICC, immunocytochemistry; IHC, immunohistochemistry; IF, immunofluorescence; ChIP, chromatin immunoprecipitation.

### Animals

B6SJL-Tg(APPSwFlLon,PSEN1*M146L*L286V)6799Vas/J transgenic (5XFAD) mice were purchased from Jackson Laboratories (Bar Harbor, ME). Five month old male 5XFAD mice were treated with RNS60 or NS (300 µl/mouse/2d) via i.p. injection for 1 month followed calcium influx assay and tissue immunostaining.

### Preparation of RNS60

RNS60 was generated at Revalesio (Tacoma, WA) using Taylor-Couette-Poiseuille (TCP) flow as described before [19], [20], [21]. Briefly, sodium chloride (0.9%) for irrigation, USP pH 5.6 (4.5–7.0, Hospira), was processed at 4°C and a flow rate of 32 mL/s under 1 atm of oxygen back-pressure (7.8 mL/s gas flow rate), while maintaining a rotor speed of 3,450 rpm. Chemically, RNS60 contains water, sodium chloride, 50–60 parts/million oxygen, but no active pharmaceutical ingredients.

**Figure 1:**
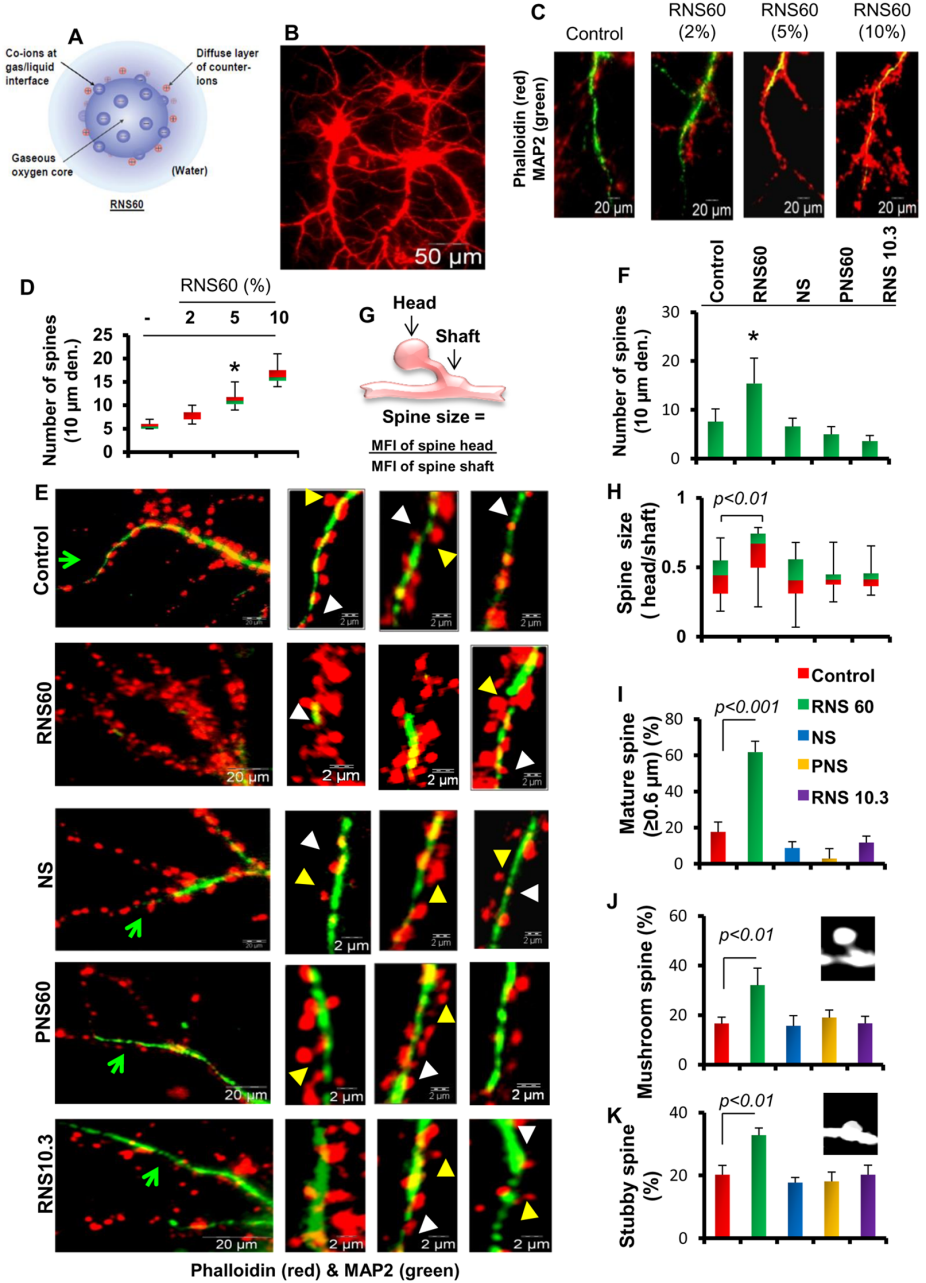
Effect of RNS60, NS, PNS60, and RNS10.3 on the number, size, and maturation of dendritic spines in hippocampal neurons. A) Schematic representation of RNS60. Three-week old hippocampal neuronal cultures (B) were treated with 2, 5, or 10% RNS60 for two days followed by immunostaining with neuronal marker MAP2 (green) and Alexa-647 conjugated phalloidin (red) for spines (C). Boxplot analyses for quantifying the spine density in neurons in response to different doses of RNS60 (D). Control-, RNS60-, NS-, PNS60-, and RNS10.3-treated neurons were double-stained with MAP2 and Phalloidin after 48 h of incubation (E). The images on the left show a larger view of dendrites, and the three images per group on the right show the spine density of dendrites collected from three separate images from each group. Yellow arrowheads indicate mushroom spines and white arrowheads indicate stubby spines. Green arrows used in the left panel are magnified and presented in one of three right side images. The spine density (F) was measured from Phalloidin-stained neurons and plotted as a function of 10-µm long dendrites (G). The cartoon shows the strategy applied to measure the spine size. (H) Accordingly, spine size was calculated from 20 images of dendrites. (I) Spines with head to neck ratio of 0.6 were considered as matured spines and their number was counted and plotted. Number of mushroom (J) and stubby (K) spines were counted from 10 different images and plotted for control-, RNS60-, NS-, RNS10.3-, and PNS60-treated hippocampal neurons.

The following controls for RNS60 were also used in this study: a) NS, unaltered normal saline from the same manufacturing batch; b) RNS10.3, normal saline that was processed through the same device without adding excess oxygen; c) PNS60, normal saline with the same final oxygen content (55±5 ppm) as RNS60 but not processed with TCP flow. Careful analysis demonstrated that all three fluids were chemically identical [19]. Liquid chromatography quadrupole time-of-flight mass spectrometric analysis also showed no difference between RNS60 and other control solutions [19]. On the other hand, by using atomic force microscopy, we studied nanobubble nucleation in RNS60 and other saline solutions and observed that RNS60 had a nanobubble composition different from that of control saline solutions [19]. This same relative pattern of nanobubble number and size was observed when positive potentials were applied to AFM surfaces with the same control solutions, suggesting the involvement of charge in stabilization of nanobubbles in RNS60 (Fig. 1A).

### Isolation and maintenance of Mouse Hippocampal neurons

Hippocampal neurons were isolated from fetuses (E18) of pregnant female mice as described by us [22], [23], [24]. Briefly, dissection and isolation procedures were performed in an ice-cold, sucrose buffer solution (sucrose 0.32 M, Tris 0.025 M; pH 7.4). The skin and the skull were carefully removed from the brain by scissors followed by peeling off the meninges by a pair of fine tweezers. A fine incision was made in the middle line around the circle of Willis and medial temporal lobe was opened up. Hippocampus was isolated as a thin slice of tissue located near the cortical edge of medial temporal lobe. Hippocampal tissues isolated from all fetal pups (n >10) were combined together and homogenized with 1 ml of Trypsin for 5 min at 37°C followed by neutralization of trypsin [23], [24]. The single cell suspension of hippocampal tissue was plated in the poly-D-lysine pre-coated 96-well and 12-well plates. Five minutes after plating, the supernatants were carefully removed and replaced with complete neurobasal media. The next day, 10 µM AraC was added to remove glial contamination in the neuronal culture. The pure cultures of hippocampal neurons were allowed to differentiate fully for 18–21 days before treatment (Fig. 1B).

### Measurement of spine density and size

For counting spine density, E18 hippocampal neurons were stained with Alexa-647 conjugated phalloidin (Cat #A22287) together with MAP2. Only densely stained neurons were counted. The total length of each dendrite was measured at 400X magnification using an Olympus BX-51 fluorescence microscope. The number of spines on the dendrites was counted under oil immersion. As some of the spines were hidden under the dendrite, only spines that protruded laterally from the shafts of the dendrites into the surrounding area of clear neuropil were counted. The spine density of a pyramidal neuron was calculated by dividing the total number of spines on a neuron by the total length of its dendrites, and was expressed as the number of spines/10 µm dendrite. The size of the dendritic spines was measured by calculating the ratio of mean fluorescent intensity (MFI) of the spine head and MFI of the dendritic shaft.

### Dendrite Visualization and Quantitative Morphometric Analysis

The length of the apical dendrite and the number of dendritic arbors were measured by tracing of MAP-2 stained neurons with Inkscape software tracing tools. All images were scaled under same color intensities. For calculating the number of collaterals, images were magnified at 100X magnification in olynpus BX51 microscope and then the number of collaterals was measured for each 100-µm long dendrites. The axon was identified by its distinct morphology and was eliminated from quantification. “Apical” dendrites were defined as the longest single protrusion, also referred to as the primary dendrite, which has the largest diameter proximal to the cell body.

### Calcium Influx Assay in Primary Mouse Hippocampal Neurons

Cultured hippocampal neurons were loaded with Fluo4-fluorescence conjugated calcium buffer (Invitrogen Molecular Probes, Cat #F10471) and incubated at 37°C for 60 min as described before [22]. Briefly, fluorescence excitation and emission spectra were recorded in a Perkin–Elmer Victor X2 Luminescence Spectrometer in the presence of 50 µM NMDA and AMPA solutions. The recording was performed with 300 repeats at 0.1 ms intervals. Oscillogram was generated with the help of Parkin Elmer 2030 software that was connected to Victor X2 calcium recorder unit. The realtime calcium noise graph was generated as a kinetic chart plotted in a logarithmic scale.

### Calcium Influx Assay in Mouse Hippocampal Slices

Male C57BL/6 animals (n = 5) were anesthetized, rapidly perfused with ice cold sterile PBS, and decapitated. The whole brain was carefully removed from the cranium. Dorsoventral slices of the hippocampus were made in a TPI PELCO 101 Vibratome series 1000 semi-automatic tissue sectioning system at a thickness of 100 micron. The slice chamber of the vibratome was filled with cutting solution (*sucrose 24.56 g, dextrose 0.9008 g, ascobate 0.0881 g, sodium pyruvate 0.1650 g, and myo-inositol 0.2703 g in 500 mL distilled water*) and continuously bubbled with 5% CO_2_ and 95% O_2_ gas mixture. The whole chamber was kept ice cold during the slicing period. Slices were then carefully transferred into Fluo-4 dye containing reaction buffer. The reaction buffer was made prior to the making of brain slices using 10 mL of artificial CSF (*119 mM NaCl, 26.2 mM NaHCO_3,_ 2.5 mM KCl, 1 mM NaH_2_PO_4,_ 1.3 mM MgCl_2,_ 10 mM glucose, bubbled with 5% CO_2_ and 95% O_2_ followed by the addition of 2.5 mM CaCl_2_*) added to one bottle of Fluo-4 dye (*Cat #F10471*), and 250 mM probenecid. Before transferring slices, a flat bottom 96 well plate (BD Falcon; Cat #323519) was loaded with 50 µL of reaction buffer per well, covered with aluminum foil, and kept in a dark place. One individual slice was placed in each well loaded with reaction buffer, and the plate was re-wrapped with aluminum foil and kept at 37°C for 20 min. The calcium assay was conducted as described above.

### Immunofluorescence analysis

Immunofluorescence analysis was performed as described earlier [25], [26], [27]. Briefly, cells cultured in 8-well chamber slides (Lab-Tek II) were fixed with 4% paraformaldehyde for 20 min followed by treatment with cold ethanol (−20°C) for 5 min and 2 rinses in PBS. Samples were blocked with 3% BSA in PBST for 30 min and incubated in PBST containing 1% BSA and rabbit anti-NR2A (1∶100), anti-GluR1 (1∶100), anti-PSD95 (1∶100) and anti-CREB (1∶100). After three washes in PBST (15 min each), slides were further incubated with cy2- and cy5-conjugated secondary antibodies (Jackson ImmunoResearch Laboratories, Inc.). For negative controls, a set of culture slides was incubated under similar conditions without the primary antibodies. The samples were mounted and observed under an Olympus IX81 fluorescent microscope. For tissue staining, brains were kept in 4% paraformaldehyde and 30-µm slices were sectioned in a cryostat followed by immunostaining as described before [28].

### Cellular Membrane Extraction

Neuronal membranes were isolated to determine the recruitment of various membrane-associated proteins to the membrane. Cells were washed with PBS, scraped into phenol-red-free HBSS and transferred to 5-mL ultracentrifuge tubes. The solution was then diluted with 100 mM sodium bicarbonate buffer (pH 11.5) and spun in an ultracentrifuge at 40,000 rpm for 1 hr at 4°C. The resultant supernatant was aspirated and the pellet was immersed in double-distilled H_2_0 and SDS and stored at −80°C overnight. The following day, the pellet was resuspended by repeated grinding and boiling.

### Immunoblot Analysis

Immunoblot analysis was carried out as described earlier [22], [29], [30], [31]. Briefly, neuronal cell homogenates were electrophoresed, proteins were transferred onto a nitrocellulose membrane, and protein band was visualized with Odyssey infrared scanner after immunolabeling with primary antibodies followed by infra-red fluorophore-tagged secondary antibody (Invitrogen, Carlsbad, CA).

### Semi-quantitative RT-PCR

Total RNA was isolated from mouse primary hippocampal neurons using Ultra spec-II RNA reagent (Biotecx Laboratories, Inc.) following manufacturer’s protocol. To remove any contaminating genomic DNA, total RNA was digested with DNase. Semi quantitative RT-PCR was carried out as described earlier [32] using a RT-PCR kit from Clontech. Briefly, 1 µg of total RNA was reverse-transcribed using oligo(dT)_12–18_ as primer and MMLV reverse transcriptase (Clontech) in a 20-*µ*l reaction mixture. The resulting cDNA was appropriately-diluted, and diluted cDNA was amplified using following primers:

nr-2a (mouse): Sense: 5′-GAGGCTGTGGCTCAGATGCTGGATT-3′

Anti-sense: 5′-GGCCCGGCTTGAGGT TTCAGAAAT G-3′

glur1 (mouse): Sense: 5′-AATGGTGGTACGACAAGGGC-3′

Anti-sense: 5′-GGATTGCATGGACTTGGGGA-3′

Amplified products were electrophoresed on a 1.8% agarose gels and visualized by ethidium bromide staining.

### Real-time PCR Analysis

It was performed using the ABI-Prism7700 sequence detection system (Applied Biosystems) as described earlier [28], [29] using primers and FAM-labeled probes from Applied Biosystems. The mRNA expressions of respective genes were normalized to the level of GAPDH mRNA. Data were processed by the ABI Sequence Detection System 1.6 software and analyzed by ANOVA.

### PCR super array analyses of plasticity-associated genes

The Mouse Synaptic Plasticity RT^2^ Profiler PCR Array (SA Biosciences; Cat #PAMM-126Z) profiles the expression of 84 key genes central to synaptic alterations during learning and memory. Briefly, mouse hippocampal neurons were treated with 10% (v/v) RNS60 and NS for 24 h followed by isolation of total RNA using Qiagen RNA isolation kit and synthesis of cDNA as described previously (26,27). Next, cDNA samples were diluted 100-fold and 2 µL of diluted cDNA was added into each well of a 96-well array plate followed by the amplification of cDNA using SYBR green technology in the ABI-Prism7700 sequence detection system. The resulting Ct value was normalized with the housekeeping gene GAPDH and then plotted in heatmap explorer software.

## Results

### RNS60 induced morphological plasticity in cultured hippocampal neurons

Since the formation and maturation of dendritic spines contribute directly to the long-term enhancement of synaptic efficacy of hippocampal neurons involved in learning and memory, we studied the effect of RNS60 on the number, size, and maturation of dendritic spines. First, we analyzed the effect of 2%, 5% and 10% v/v RNS60 on the spine density. Interestingly, RNS60 dose-dependently increased the density of dendritic spines in cultured hippocampal neurons (Fig. 1C–D). A detailed morphological analyses further revealed that RNS60, but not controls such as NS, PNS, and RNS10.3, stimulated the number (Fig. 1E–F), size (Fig. 1G–H), and maturation (Fig. 1J–K) of dendritic spines in hippocampal neurons. This suggests that RNS60 enhances the synaptic maturation of hippocampal neurons by enriching the density and size of dendritic spines.

**Figure 2:**
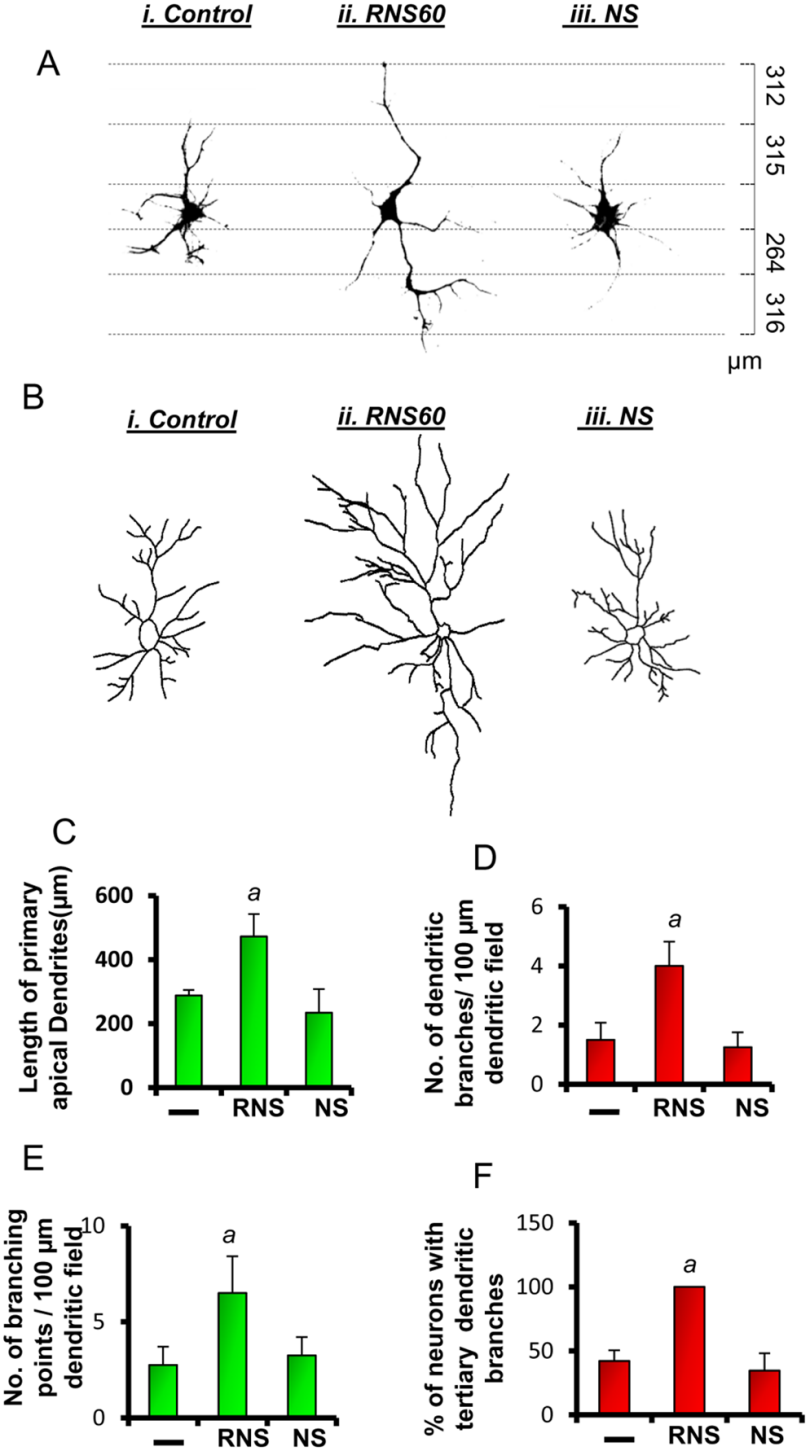
RNS60 stimulates the dendritic length, and the number of collaterals of primary dendrites in cultured hippocampal neurons. (A, B) Hippocampal neuronal cultures were treated with 10% RNS60 or NS for two days followed by immunostaining with the dendritic marker MAP2. Stained neurons were traced in scalable vector graphics (SVG) software inkscape for only primary dendrites (A) and for detailed branching (B). (C) The length of primary dendrites, (D) number of collaterals per 100 µm dendrite, (E) branching points, and (F) tertiary branches (plotted in a percent scale to RNS60) were calculated from twenty images of each treatment group. *^a^p<0.01 versus control.*

The shape, size, and complexity of dendritic arbors are also associated with long-term synaptic facilitation [33], [34] in hippocampal neurons. Therefore, we tested the effect of RNS60 on enlargement of apical dendrites, formation of new arbors, and number of neurons with tertiary branches. Our tracing analyses (n = 10 per group) clearly showed that RNS60 significantly increased the elongation of primary dendrites (Fig. 2A & 2C), the number of dendritic collaterals (Fig. 2B & 2D), and the number of neurons with tertiarydendritic branches (Fig. 2E–F).

### RNS60 stimulated inward calcium currents in cultured hippocampal neurons

**Figure 3:**
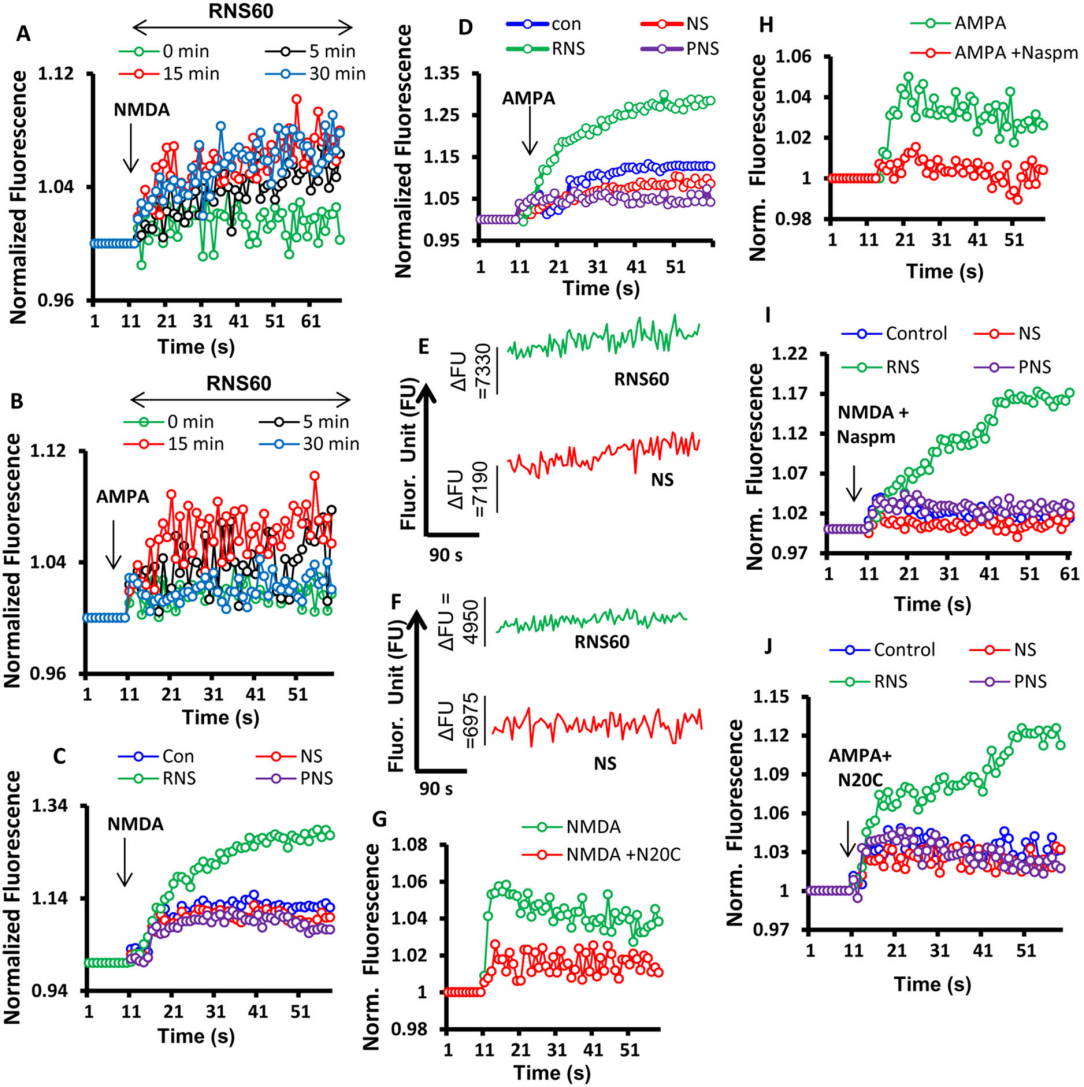
Effect of RNS60, PNS60, and NS on NMDA and AMPA-dependent calcium influx in cultured mouse hippocampal neurons. Mouse hippocampal neurons were treated with10% (v/v) RNS60 for 5, 15, or 30 minutes under serum free condition followed by treatment with 50 µM NMDA and AMPA as described in the materials and methods section. (A) Normalized fluorescence value of NMDA-dependent and (B) AMPA-dependent calcium influx monitored for 300 repeats over a 90-second period of time in cultured hippocampal neurons. NMDA-dependent (C) and AMPA- dependent (D) calcium influx in primary neurons after 24 h of RNS60, NS, or PNS60 treatment. The data are presented as the mean of three independent experiments. Noise recordings of (E) NMDA-driven and (F) AMPA-driven calcium currents in RNS60 and NS-treated primary neuronal cultures. Mouse hippocampal neurons were treated with RNS60 for 24 hrs followed by the incubation with either 20 µM of NMDA receptor inhibitor N20C or 50 µM of AMPA receptor inhibitor Naspm for five minutes. After that Cells were treated with 50 µM NMDA (G) or AMPA (H) followed by calcium influx measurement. To nullify the secondary involvement of AMPA receptor in NMDA-dependent calcium currents, cells were treated with NMDA together with Naspm followed by the recording of calcium influx (I). Similarly AMPA-dependent calcium influx was measured in the presence of N20C. Results are presented as the mean of three independent experiments. FU = fluorescence unit.

Since an increase in the number of matured spines directly regulates synaptic transmission in the post-synaptic neurons via NMDA and AMPA-regulated ionotropic receptors, our next aim was to study calcium influx through NMDA and AMPA receptors after RNS60 treatment. As the activation of ionotropic glutamate receptors is a very rapid and transient process, we first measured calcium influx during short time periods after RNS60 treatment in cultured mouse hippocampal neurons. Incubation of the cells with RNS60 for 5, 15, and 30 minutes did not induce a strong induction of NMDA- (Fig. 3A) or AMPA- (Fig. 3B) dependent calcium influx, even though in all cases, RNS60 showed high amplitude oscillations, indicating that the excitability of ionotropic glutamate receptors was not altered. Next, we examined the effect of RNS60 on NMDA- and AMPA-dependent calcium influx in cultured hippocampal neurons after 24 h of incubation. Interestingly, we observed that RNS60, but neither NS nor PNS60, now stimulated calcium influx in the presence of NMDA (Fig. 3C) or AMPA (Fig. 3D). Moreover, prolonged incubation of hippocampal neurons with RNS60 resulted in high frequency calcium influx in the presence of NMDA (Fig. 3E) or AMPA (Fig. 3F), suggesting that RNS60 may potentiate postsynaptic membrane depolarization, eventually leading to the long term enhancement of synaptic activity [35] in hippocampal neurons. Moreover, the induction of calcium current through NMDA and AMPA receptors are known to be influenced by a passive or secondary activation of AMPA and NMDA receptors, respectively. Therefore, in order to nullify the secondary involvement of NMDA activation in AMPA current, we used a specific NMDA receptor blocker *N20C*. Similarly to reject the passive involvement of AMPA receptor in NMDA activation, we also used a specific AMPA blocker *Naspm*. We observed that a shorter pre-incubation with 20 µM of N20C and 50 µM of Naspm efficiently knocked down the NMDA-(Fig. 3GI) and AMPA-induced (Fig. 3H) calcium currents in cultured hippocampal neurons. Next, we recorded the NMDA-driven calcium currents in the presence of AMPA-antagonist Naspm (Fig. 3I) and AMPA-driven (Fig. 3J) calcium currents in the presence of N20C in RNS-, NS-, and PNS-treated hippocampal neurons. In both cases, the recorded current showed significant induction in RNS60-treated, but neither NS- nor PNS-treated neurons, nullifying the contribution of passive calcium currents.

### RNS60 modulated the expression of plasticity-associated genes in hippocampal neurons

**Figure 4:**
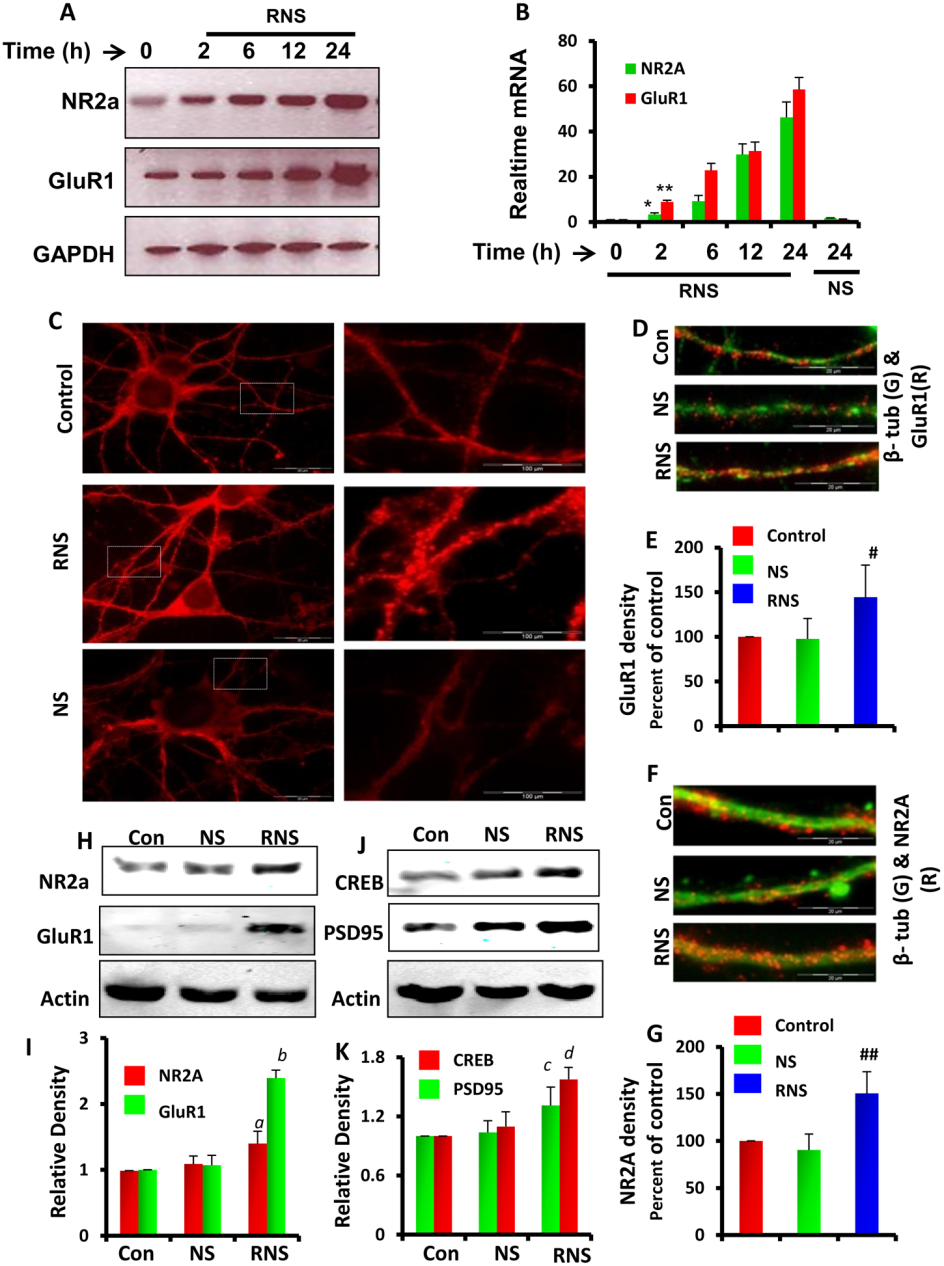
Effect of RNS60 on the expression of plasticity-associated proteins in mouse hippocampal neurons. (A) RT-PCR and (B) real-time PCR analyses of NR2A and GluR1 genes were performed in mouse primary hippocampal neurons at 0, 2, 6, 12, and 24 h of RNS60 (10% v/v) treatment. Results are mean ± SD of three independent results. *p<0.001 versus control-NR2A; **p<0.001 versus control-GluR1. (C) Immunofluorescence analysis of PSD95 in mouse hippocampal neurons after 24 hrs of RNS60 or NS treatment as described in the materials and methods section. The panels on the right are magnified views of the dotted boxes in the panels on the left. (D) Dual immunofluorescence analysis of GluR1 (red) and beta tubulin (green) in mouse primary neurons treated with RNS60 and NS for 24 hrs. (E) Numbers of GluR1-immunoreactive spines were counted in 50-micron long neurites of control, NS-, and RNS60-treated hippocampal neurons and then plotted as percent compared to untreated controls. Results are mean ± SD of three independent results. *^#^p<0.01 versus control*. (F) Double labeling of NR2A (red) and beta tubulin (green) in mouse hippocampal neurons treated with RNS60 or NS for 24 hrs. (G) Numbers of NR2A-immunoreactive spines were plotted as percent of control in control, NS-, and RNS60-treated neurons. Results are mean ± SD of three independent results and *^##^p<0.001 versus control*. Mouse primary neurons were treated with RNS60 and NS for 24 hrs followed by immunoblot analyses of NR2A and GluR1 (H); CREB and PSD-95 (J). (I and K) Representative histograms are relative densitometric plots of respective immunoblot analyses. *^a^p<0.01 versus control NR2A, ^b^p<0.001 versus control GluR1, ^c^p<0.01 versus control CREB, and ^d^p<0.01 versus control PSD95*. Results are mean ± SD of three independent experiments.

Because RNS60 failed to significantly induce NMDA- and AMPA-dependent calcium influx after short-term incubation, we assume that RNS60 is not involved in the transient phosphorylation of NMDA and AMPA receptor subunits. However, the induction of NMDA- and AMPA-dependent calcium influx after 24 h of incubation with RNS60 prompted us to investigate the effect of RNS60 on the expression of plasticity-associated genes in cultured hippocampal neurons. Time-dependent mRNA analysis showed that RNS60 increased NR2A and GluR1 expression levels within 2 h of treatment (Fig. 4A–B). The level of upregulation of both NR2A and GluR1 mRNA continued to increase with time throughout the duration (24 h) of the experiment (Fig. 4A–B). We corroborated our mRNA expression studies with protein expression analysis of NR2A, GluR1, PSD95, and CREB in hippocampal neurons. Immunofluorescence analysis of PSD95 (Fig. 4C), GluR1 (Fig. 4D–E), and NR2A (Fig. 4F–G) showed that RNS60 strongly upregulated these proteins in the projections of hippocampal neurons (Fig. 4C–G). Immunoblot analyses of NR2A and GluR1 (Fig. 4H–I) along with CREB and PSD95 (Fig. 4J–K) further confirmed that RNS60 significantly stimulated the expression of multiple plasticity-related proteins in hippocampal neurons.

**Figure 5:**
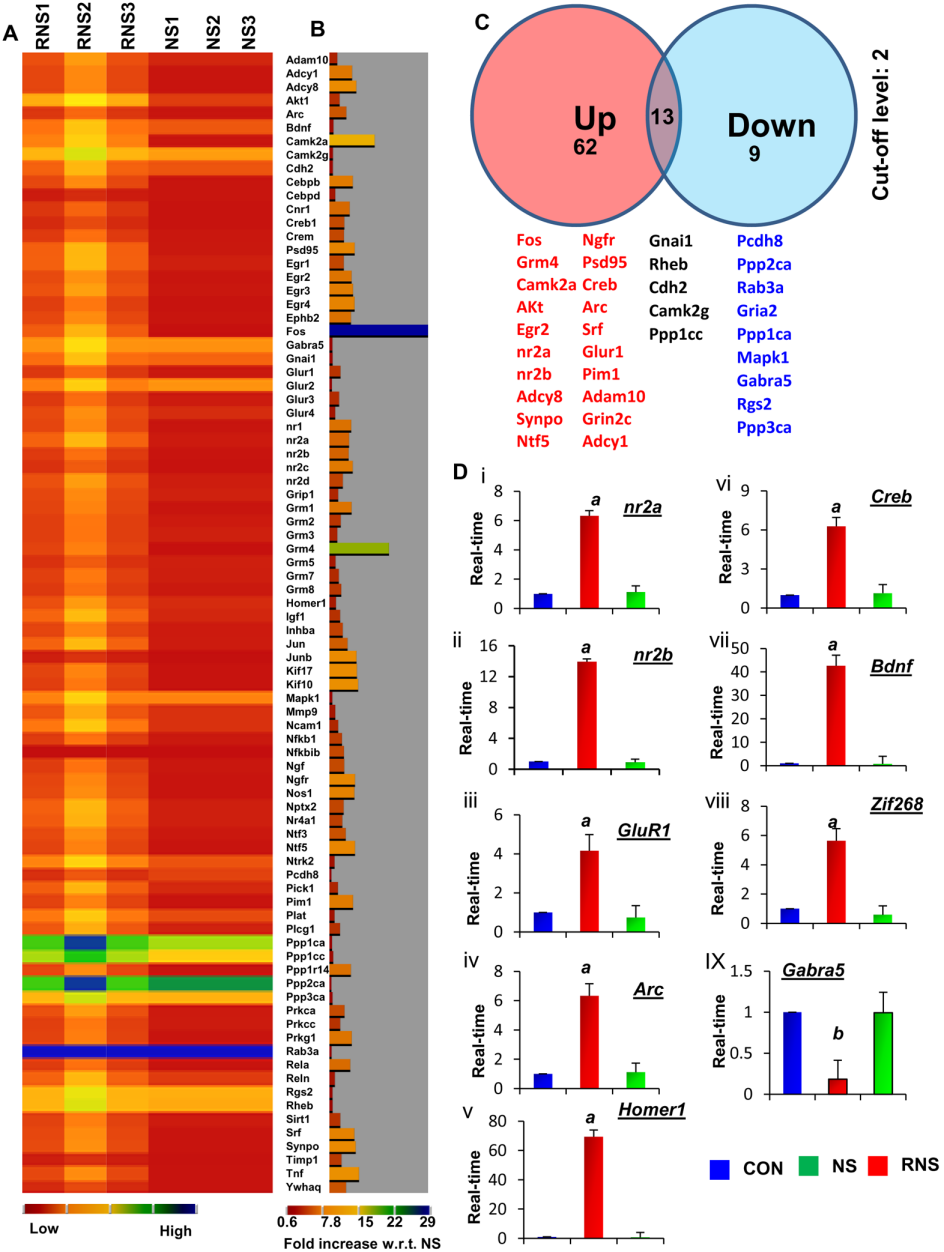
Effect of RNS60 on the expression of plasticity-associated genes in cultured mouse hippocampal neurons. Mouse primary neurons were treated with 10% RNS60 or NS for 24 h followed by the analysis of plasticity-associated gene expression from total mRNA by mRNA-based super array technology. (A) Heatmap expression profile of 84 plasticity-associated genes as derived from mRNA-based array. Red represents the minimum and blue represents the maximum level of expression. (B) Histogram summary of the expression of all genes shown in the heatmap. (C) Venn diagram summarizing the list of genes that were upregulated, downregulated, and unaltered by RNS60. (D) Real time mRNA analysis of eight randomly selected genes including NR2A (i), NR2B (ii), GluR1 (iii), Arc (iv), Homer1 (v), CREB (vi), BDNF (vii), Zif-268 (viii), and Gabra5 (ix) in mouse hippocampal neurons under similar treatment conditions. Results are mean ± SD of three independent experiments. *^a^p<0.001 versus control*; *^b^p<0.01 versus control-Gabra5.*

Neuronal plasticity is controlled by multiple proteins. To investigate which other plasticity-associated genes are affected by RNS60 in the hippocampus, we performed mRNA-based super array analysis of plasticity-related genes in both RNS60- and NS-treated cultured hippocampal neurons. The results are summarized in a heat-map presentation (Fig. 5A–B). We observed that 62 of 84 analyzed genes were upregulated, 9 genes were down-regulated, and 13 genes remained unaltered in RNS60-treated hippocampal neurons as compared to NS-treatment (Fig. 5C). Among the upregulated genes, we identified IEGs including *arc*, *zif-268*, and *c-fos*; synapse-associated genes including *synpo*, *adam-10*, and *psd-95*; and most interestingly genes encoding NMDA receptor subunits including *nr1*, *nr2a*, *nr2b,* and *nr2c*; genes for the AMPA receptor subunit *glur1*; and genes for neurotrophic factors and their receptors including *bdnf*, *nt3*, *nt5, and ntrk2*. Furthermore, CREB is an important molecule for plasticity as it controls the transcription of various plasticity-related molecules [36], [37]. It is interesting to see that RNS60 upregulates CREB as well as different signaling molecules that are involved in the activation of CREB. For example, the adenylate cyclase pathway is known to activate CREB via the cAMP – protein kinase A (PKA) pathway [38]. RNS60 treatment increases the expression of genes encoding for different adenylate cyclases (*adcy1* and *adcy8*) in mouse hippocampal neurons as compared to NS treatment (Fig. 3A–B). CREB is also activated by Ca^2+^/calmodulin-dependent protein kinase II (CAM kinase II) and Akt [38], [39], and RNS60 also upregulated the expression of *camk2a* and *akt1* (Fig. 3A–B). In contrast, RNS60 treatment down-regulated the expression of *Gria2*, *Ppp1ca*, *Ppp2ca*, and *Ppp3ca*, proteins encoded by genes that are known to support long-term depression (Fig. 5A–C).

In order to validate the array-based mRNA results, we performed quantitative real-time PCR analysis of nine randomly chosen genes from the list. We confirmed that RNS60 indeed upregulated the mRNA expression of *nr2a* (Fig. 5Di), *nr2b* (Fig. 5Dii), *glur1* (Fig. 5Diii), *arc* (Fig. 5Div), *homer-1* (Fig. 5Dv), *creb* (Fig. 5Dvi), *bdnf* (Fig. 5Dvii), and *zif-268* (Fig. 5Dviii) by several fold in hippocampal neurons as compared to untreated neurons. In contrast, RNS60 inhibited the mRNA expression of *Gabra5* (Fig. 5Dix). These results were specific as NS-treatment did not alter the expression of these genes (Fig. 5D).

### RNS60 upregulated plasticity-associated genes and stimulated calcium influx in primary mouse hippocampal neurons via phosphatidylinositol 3-kinase (PI3K)

**Figure 6:**
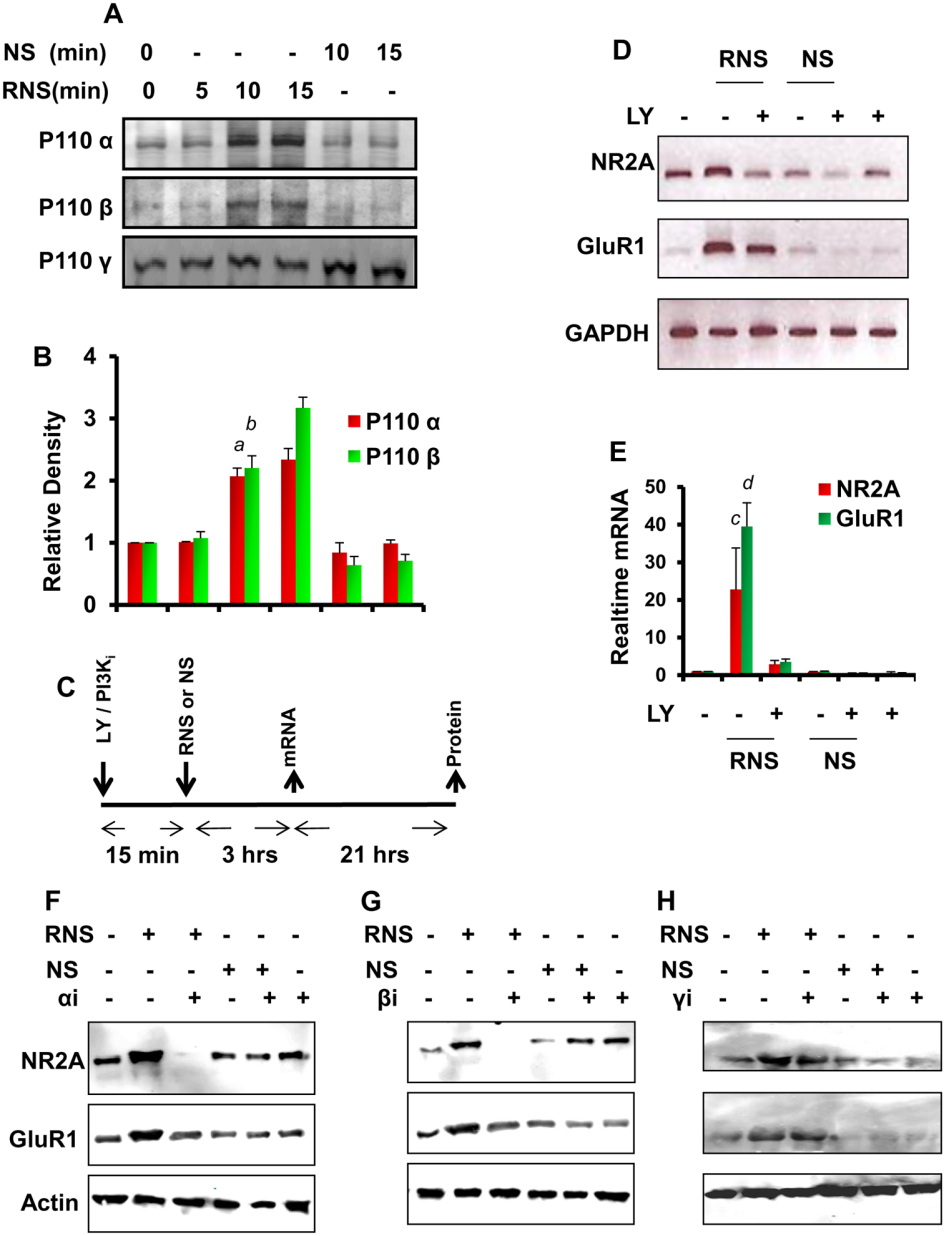
The role of the PI3K pathway in RNS60-mediated upregulation of plasticity-associated genes in mouse hippocampal neurons. (A) Mouse hippocampal neurons were stimulated with RNS60 or NS for 5, 10, 15, and 30 minutes under serum-free condition followed by immunoblot analyses of p110 α, β, and γ in membrane fractions. (B) Relative densitometric analysis of p110 α and β bands. Results are mean ± SD of three independent experiments. *^a^p<0.001 versus control-p110α; ^b^p<0.001 versus control-p110β*. (C) Cells pretreated with 2 µM LY294002 for 15 minutes were stimulated with 10% RNS60. After 3 h of stimulation, the mRNA expression of NR2A and GluR1 was analyzed by semi-quantitative RT-PCR (D) and real-time PCR (E). After 24 hrs of treatment with 5 µM of p110α (F), 2 µM of p110β (G), and 20 µM of p110γ (H) inhibitors RNS60-stimulated neurons were immunoblotted for NR2A and GluR1 proteins. Results are mean ± SD of three independent experiments. *^c^p<0.001 versus control-NR2A; ^d^p<0.001 versus control-GluR1*.

Next we investigated the mechanisms by which RNS60 increased these plasticity related genes in cultured hippocampal neurons. Recently we have observed that RNS60 activates PI3K in microglial cells [19]. Because PI3K is linked to a diverse group of cellular functions, we examined whether activation of PI3K by RNS60 played a role in RNS60-mediated stimulation of plasticity. At first, we tested the effect of RNS60 on PI3K activation in hippocampal neurons. Class IA PI3K, which is regulated by receptor tyrosine kinases, consists of a heterodimer of a regulatory 85-kDa subunit and a catalytic 110-kDa subunit (p85:p110α/β/δ). Class IB PI3K, on the other hand, consists of a dimer of a 101-kDa regulatory subunit and a p110γ catalytic subunit (p101/p110γ). While in resting condition, subunits of PI3K are located mainly in cytoplasm, upon activation, these are translocated to the plasma membrane [40], [41]. Therefore, we monitored the activation of class IA and IB PI3K by the recruitment of p110α, p110β and p110γ to the membrane. Western blotting of membrane fractions for p110 subunits suggests that RNS60 specifically induces the recruitment of p110α and p110β, but not p110γ, to the membrane (Fig. 6A). Densitometric analysis of the p110α and p110β at different time points of RNS60 stimulation indicates significant activation of PI3K at 10 and 15 min (Fig. 6B). On the other hand, we did not observe any activation of p110α and p110β PI3K at 5 min of RNS60 stimulation (Fig. 6A–B). Again these results were specific as NS remained unable to activate p110α and p110β PI3K at either 10 or 15 min of RNS60 stimulation. Together, these results suggest that RNS60 activates type IA PI3K p110α and p110β, but not type IB PI3K p110γ, in hippocampal neurons.

Next, to understand whether modulation of PI3K signaling pathway is involved in the RNS60-induced neuronal plasticity, we pretreated primary mouse hippocampal neurons with 2 µM PI3K inhibitor (LY294002) for 15 min followed by stimulation with 10% RNS60 or NS. After 3 h of stimulation, mRNA expression of NR2A and GluR1 was monitored by RT-PCR and real-time PCR. In this instance as well, RNS60 treatment increased the expression of NR2A and GluR1 (Fig. 6C–E). However, LY294002 abrogated RNS60-mediated increase in NR2A and GluR1 expression in hippocampal neurons (Fig. 6C–E). However, LY29402 inhibits the activation of both class 1A and 1B PI3K. Therefore, our next aim was to identify the specific class of PI3K that was involved in the RNS60-mediated upregulation of NR2A and GluR1 in hippocampal neurons. We used three different PI3K inhibitors: GDC-0941 (an inhibitor of p110α); TGX-221 (an inhibitor of p110β); and AS-605240 (an inhibitor of p110γ). Interestingly, the pretreatment of α and β inhibitors, but not γ inhibitor, significantly suppressed the RNS60-stimulated expression of NR2A and GluR1 in cultured hippocampal neurons (Fig. 6F–H) suggesting that class 1A, not class 1B PI3K, is involved in the upregulation of plasticity-associated genes in RNS60-stimulated neurons.

**Figure 7:**
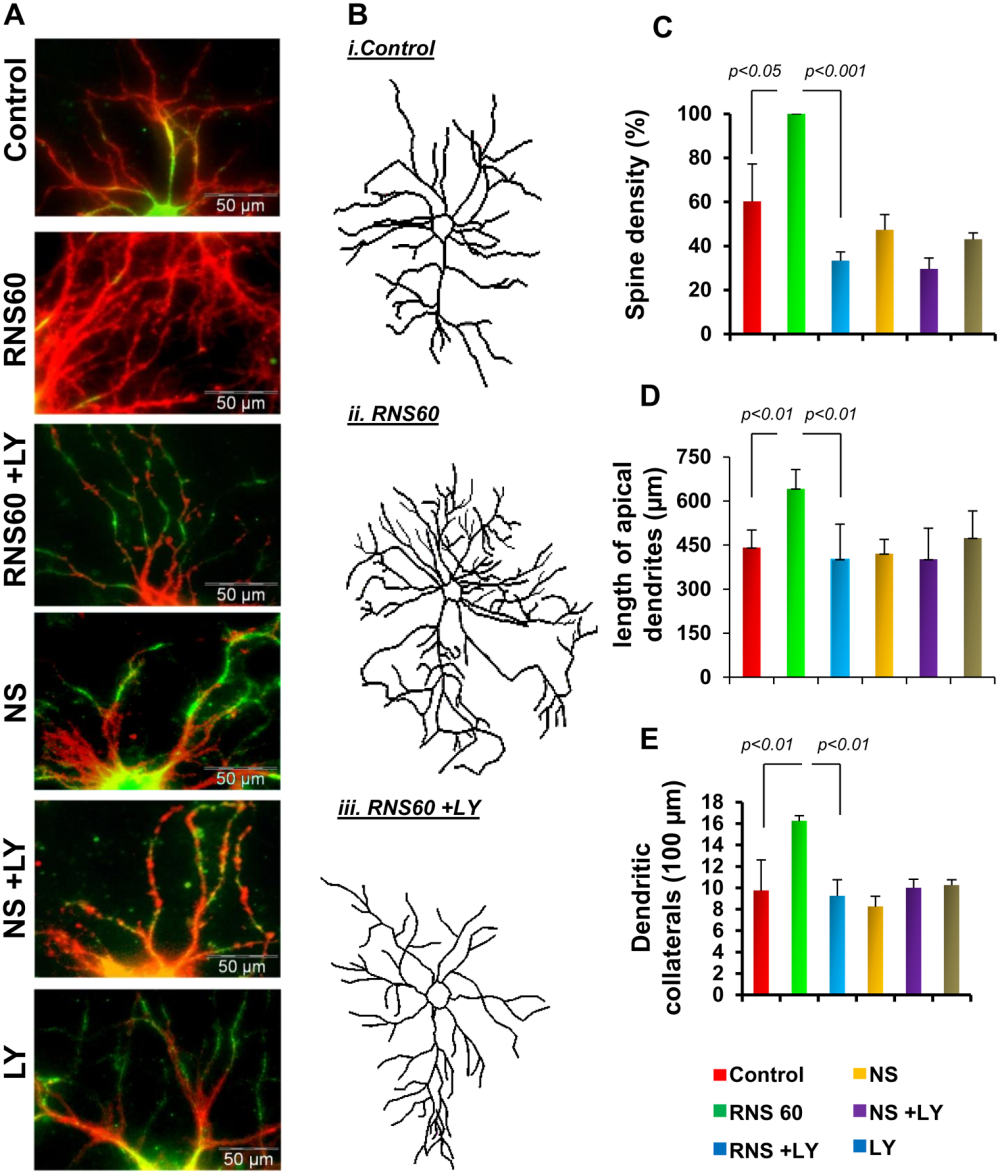
Activation of PI3K regulates morphological plasticity in RNS60-treated mouse hippocampal neurons. (A) LY294002 pre-treated mouse hippocampal neurons were stimulated with RNS60 and NS for 48 hrs followed by double-immunostaining with MAP2 (green) and Phalloidin (red) to demonstrate the spine density. (B) Neurons were traced by Inkscape software after 48 hrs of treatment with RNS (ii) and NS (iii). (C) Spine density, (D) dendritic length, and (E) dendritic branches were measured from 10 different neurons of each treatment group.

Since reduced expression of NR2A and GluR1 is linked to the decreased spine density and dendritic maturation of neurons, we tested the role of the PI3K pathway in the RNS60-mediated increase in spine density and dendritic morphologies. A 15-min pretreatment with 2 µM LY29402 significantly decreased the RNS60-induced increase in spine density in hippocampal neurons (Fig. 7A), as quantified by spine density counts (Fig. 7C). Interestingly, LY29402 also attenuated primary dendritic length and number of dendritic collaterals in RNS60-treated neurons (Fig. 7Bi–iii), as quantified in figure 7D–E.

**Figure 8:**
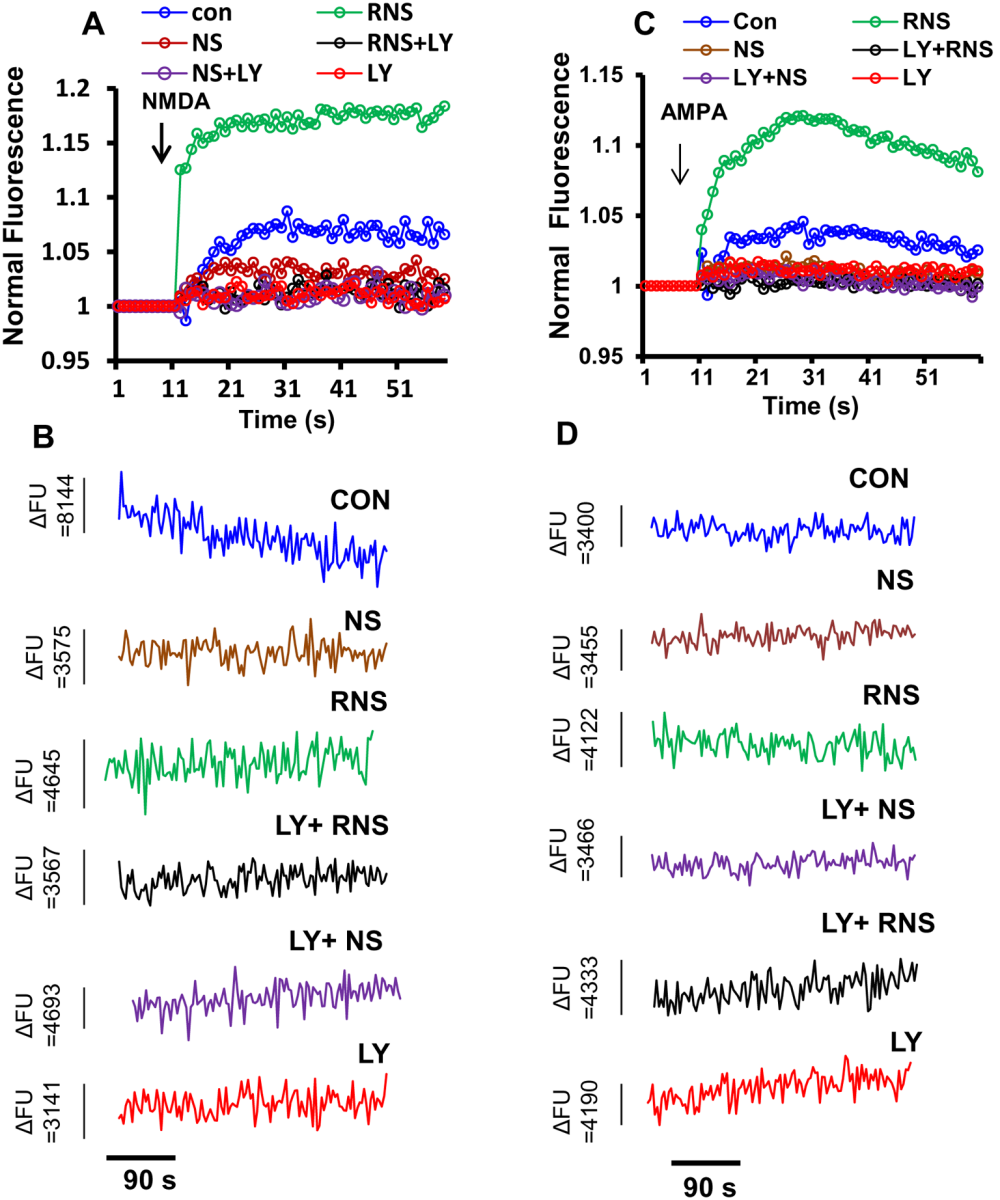
Activation of PI3K regulates both NMDA- and AMPA-sensitive calcium influx in RNS60-treated mouse hippocampal neurons. Mouse hippocampal neurons pretreated with 2 µM LY294002 for 15 minutes were incubated with 10% (v/v) RNS60 for 24 h under serum free condition followed by the measurement of calcium influx in the presence of 50 µM NMDA (A) and AMPA (B). Representative images are (C) NMDA- and (D) AMPA-mediated noise recordings of calcium influx in control, NS-, RNS60-, (RNS60+LY)-, and LY-treated primary hippocampal neurons. Results are mean of three independent experiments. FU = fluorescence unit.

The critical event leading to the induction of synaptic transmission appears to be the influx of calcium currents into the postsynaptic spine. Therefore, we analyzed the effect of LY294002 on RNS60-induced calcium influx. As shown earlier, RNS60 treatment stimulated calcium influx in the presence of either NMDA (Fig. 8A–B) or AMPA (Fig. 8C–D). However, LY294002 ablated the stimulatory effect of RNS60 on NMDA- (Fig. 8A–B) and AMPA-induced (Fig. 8C–D) calcium influx. Together, these data suggest that RNS60’s effects on plasticity in hippocampal neurons are mediated via activation of the PI3K pathway.

### RNS60 treatment increases the expression of plasticity-associated proteins in vivo in the hippocampus of 5XFAD transgenic mice

**Figure 9:**
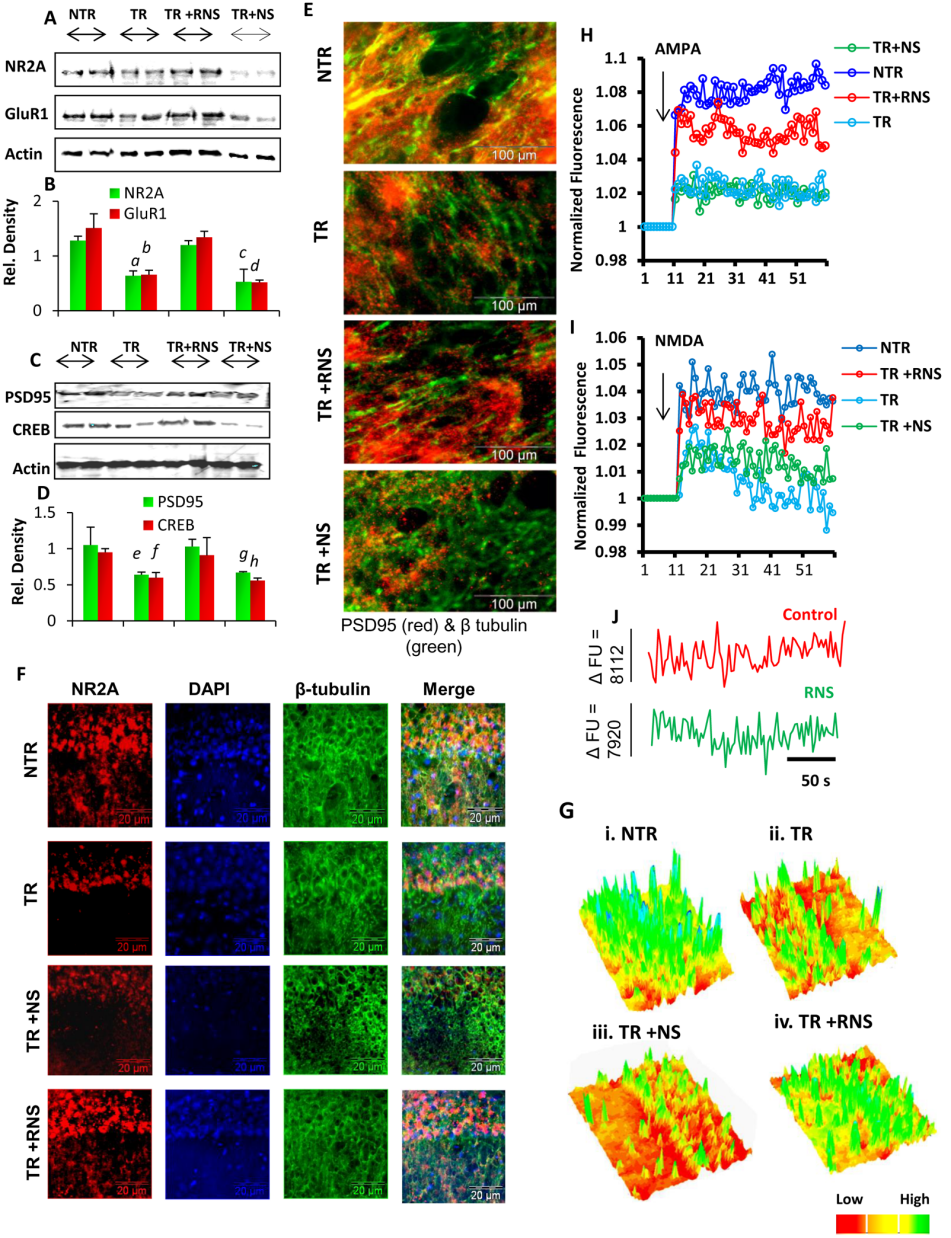
Effect of RNS60 on the expression of plasticity-associated molecules *in vivo* in the hippocampus of 5XFAD transgenic animals. Five-month old transgenic mice (n = 5 per group) were injected i.p. with RNS60 or NS (300 µL/mouse/2d) for 60 days. After that, animals were sacrificed and their hippocampi were analyzed for the expression of different plasticity-associated proteins. Immunoblot analysis of GluR1 and NR2A (A); PSD-95 and CREB (C) in the hippocampal extracts of NTR (non-transgenic), TR (transgenic), TR+NS, and TR+RNS animals. Relative densitometric analyses of GluR1 and NR2A (B) & PSD95 and CREB (D). Results are mean ± SEM of five mice per group. *^a^p<0.001 versus control-GluR1; ^b^p<0.005 versus control-NR2A; ^c^p<0.001 versus TR-GluR1; ^d^p<0.005 versus TR-NR2A;^ e^p<0.005 versus control-PSD95;^ f^p<0.001 versus control-CREB;^ g^p<0.001 versus TR-PSD95;^ h^p<0.005 versus TR-CREB*. (E) Hippocampi of NTR and TR animals treated with RNS60 or NS were stained with PSD95 (red) and beta-tubulin (green). Representative images show the distribution of PSD95 in the presynaptic branches of CA1 nucleus. (F) Double labeling of NR2A (red) and beta tubulin (green) in CA-1 hippocampus of NTR-, TR-, (TR+RNS60)-, and (TR+NS)- animals. (Gi–iv) The distribution of NR2A in the CA-1 nucleus is shown in a 3D contour diagram as signal hotspot (Image Dig software). Red, yellow, green, and blue colors indicate regions with less, moderate, high, and very high distribution of NR2A receptors, respectively. (H) AMPA- and (I) NMDA-dependent calcium currents were measured in the hippocampal slices of NTR, TR, (TR+RNS60), and (TR+NS) animals as described under materials and methods. (J) Representative noise recordings of calcium currents in hippocampal slices of NTR and (TR+RNS60) mice. FU = fluorescence unit.

Strong down-regulation of NMDA and AMPA receptor proteins and loss of calcium excitability in hippocampal neurons are often observed in AD brain, and a reversal of these cellular events may have implications in AD and other forms of dementia. Although at present, we do not know how RNS60 may enter into the CNS, we have observed activation of type 1A PI-3 kinase, RNS60-specific signaling event, in the nigra of mice within only 3 h of intraperitoneal administration, suggesting that RNS60 may enter into the CNS [21]. Accordingly, peripheral administration of RNS60 protected dopaminergic neurons in the nigra of MPTP-intoxicated mice [21]. In another study, RNS60 treatment also protected mice from relapsing-remitting experimental allergic encephalomyelitis [20]. Therefore, next we wanted to study the effect of RNS60 treatment on the expression of these hippocampal proteins in 5XFAD mice, an accelerated model of AD. First, we performed immunoblot analyses of different hippocampal proteins in 5XFAD transgenic (TR) and age-matched non-transgenic (NTR) mice, as well as in transgenic animals treated with RNS60 (TR+RNS60) or NS (TR+NS). Immunoblot analysis revealed a strong down-regulation of ionotropic glutamate receptor subunits including NR2A and GluR1 (Fig. 9A–B), and other plasticity-associated proteins including PSD-95 and CREB (Fig. 9C–D), in the hippocampus of TR mice as compared to NTR mice. This deficit was almost completely restored by the treatment with RNS60, while NS remain ineffective. Consistently, immunofluorescence analysis showed that RNS60 treatment significantly upregulated the expression of PSD95 (Fig. 9E) and NR2A (Fig. 9Fi–iv) in the hippocampus of TR animals. Of note, the number of signal hotspots in representative 3D intensity plot of RNS60-treated TR mice was similar to that of NTR mice (Fig. 9Gi–iv).

Next, we examined whether we could record calcium influx in hippocampal slices of adult mice. Consistent with the decreased expression of plasticity-associated molecules in the hippocampus of TR mice as compared to NTR mice, AMPA- (Fig. 9H) and NMDA-dependent (Fig. 9I) calcium influx was also reduced in hippocampal slices of TR mice compared to NTR mice. However, AMPA- and NMDA-dependent calcium influx increased in hippocampal slices of TR mice after RNS60 treatment (Fig. 9H–I). Interestingly, the level of calcium influx in hippocampal slices of (TR+RNS) group was comparable to that observed in hippocampal slices of the NTR group. As evident from Figure 9J, RNS60 treatment evoked oscillatory amplitude in the hippocampus of TR mice to a level that is similar to untreated NTR mice.

## Discussion

It is believed that neuronal plasticity decreases with old age and in patients with AD. Therefore, exploring ways to boost plasticity in AD or aging is an important area of research. Although there are other drugs and approaches for improving brain function, here we introduce a simple saline-based agent to augment plasticity. RNS60 is generated by subjecting normal saline to Taylor-Couette-Poiseuille (TCP) turbulence in the presence of elevated oxygen pressure. It does not contain any active pharmaceutical ingredient [19], [20]. Due to TCP turbulence, RNS60 is proposed to contain charge-stabilized nanostructures consisting of an oxygen nanobubble core surrounded by an electrical double-layer at the liquid/gas interface [19], [20].

Here, we delineate the first evidence that saline generated due to TCP turbulence is capable of improving plasticity in cultured hippocampal neurons and *in vivo* in the hippocampus of 5XFAD transgenic mice. Our conclusion is based on the following: *First,* we observed that RNS60 induced the number, size, and maturation of dendritic spines in cultured hippocampal neurons, suggesting a beneficial role of RNS60 in regulating the synaptic efficacy of neurons. *Second,* RNS60 increased the dendritic length and collaterals in neurons further corroborating the role of RNS60 in stimulating the morphological plasticity of neurons. *Third,* we observed that RNS60 did not alter the calcium dependent excitability of hippocampal neurons, but rather stimulated inbound calcium currents in these neurons through ionotropic glutamate receptor. This indicates that RNS60 modulates plasticity-related activities. *Fourth,* RNS60 induced the expression of a broad spectrum of plasticity-associated molecules in hippocampal neurons. *Fifth,* RNS60 augmented the levels of several genes, proteins of which stimulate signaling pathways (adenylate cyclase, CAM kinase II and Akt) for the activation of CREB, the master regulator of plasticity. *Sixth,* proteins encoded by several genes such as *Gria2*, *Ppp1ca*, *Ppp2ca*, and *Ppp3ca* are known to support long-term depression [42]. It is interesting to see that RNS60 down-regulated the expression of *Gria2*, *Ppp1ca*, *Ppp2ca*, and *Ppp3ca* in hippocampal neurons. *Seventh,* RNS60 treatment increased the expression of plasticity-associated molecules and augmented calcium influx *in vivo* in the hippocampus of 5XFAD transgenic mice. These results together clearly demonstrate that RNS60 effectively increased neuronal plasticity and thus may have prospects as a therapeutic agent in patients with AD and other dementias.

A growing body of evidence suggests that the excessive activation of glutamate-operated NMDA receptors in postsynaptic neurons is the primary factor of progressive neuronal loss in AD [35]. Different noncompetitive and uncompetitive NMDA receptor blockers are being used for the treatment of AD [43]. However prolonged use of these drugs eventually destroys the normal excitability of these receptors, which is essential for the viability of these neurons. Moreover, these specific inhibitors of NMDA receptors generate a wide range of side effects including chest pain, nausea, increased heart rate, breathing trouble, lowered urination, and different digestive disorders because of their poor metabolic clearance among older populations [44], [45]. In contrast, RNS60 is chemically identical to isotonic saline with additional oxygen. In our study, RNS60 treatment generated high amplitude NMDA-dependent calcium oscillations both in cell culture and *in vivo* experiments. Since high amplitude calcium wave corresponds to the excitability of ionotropic receptors, we can infer that RNS60 does not alter the normal excitability of NMDA receptors. Moreover, it induced the expression many growth supportive molecules including CREB, BDNF and NTRs, which are required for the survival of neurons; synaptic proteins including PSD95, ADAM-10, and Synpo, which are required for the maintenance of synaptic structure; receptor proteins including NR2A, GluR1, and NR2B, which are needed for calcium excitability of the postsynaptic neurons; and IEGs such as c-FOS, Arc, Homer 1, and Zif-268 essential for neuroplasticity, leading to memory consolidation [46], [47], [48].

Signaling mechanisms leading to plasticity are becoming clear. Recently, we and others have reported that the master regulator cAMP response element-binding (CREB) plays an important role in plasticity and several plasticity-associated genes contain multiple cAMP response elements (CRE) in their promoter regions [22], [49], [50], [51], [52]. Recently, we have also demonstrated that RNS60 induces the activation of CREB in microglial cells via type IA phosphatidylinositol 3-kinase (PI3K) in microglial cells [19]. PI3K is a key signaling molecule implicated in the regulation of a broad array of biological responses including cell survival [41]. For class IA PI3K, the p85 regulatory subunit acts as an interface by interacting with the IRS-1 through its SH2 domain and thus recruits the p110 catalytic subunit (p110α/β) to the cell membrane, which in turn activates downstream signaling molecules like Akt/protein kinase B and p70 ribosomal S6 kinase [41]. On the other hand, for class IB PI3K, p110γ is activated by the engagement of G-protein coupled receptors. The p110γ then catalyzes the reaction to release phosphatidylinositol (3,4,5)-triphosphate as the second messenger, using phosphatidylinositol (4,5)-bisphosphate as the substrate, and activates downstream signaling molecules [40]. Here we demonstrate that RNS60 induces the activation of both the subunits of type IA PI-3K (p110α and p110β) without modulating type IB PI-3K p110γ in primary hippocampal neurons, suggesting the specific activation of type IA p110α/β PI3K in neurons. Accordingly, selective knock-down of type 1A, but not type 1B, receptor ameliorated the RNS60-stimulated expression of plasticity-associated genes. Moreover, decrease of spine density, and other morphological features and stimulation of calcium influx in hippocampal neurons by inhibitors of PI3K suggest that RNS60 increases long term synaptic efficacy via PI3K.

Due to the unavailability of proper detection techniques to track and detect nanobubbles, at present, we do not have any direct way to measure RNS60 within the brain. However, recently we have found that within 3 hours of intraperitoneal administration, RNS60 induces the activation of class 1A PI3K and the upregulation of IκBα, signature events of RNS60, *in vivo* in the nigra [31]. Therefore, it is possible that RNS60 enters the brain.

In summary, we have demonstrated that RNS60 treatment upregulates plasticity-associated molecules and calcium influx in cultured hippocampal neurons and *in vivo* in the hippocampus of 5XFAD mice. In healthy human beings and asthma patients, phase I testing has been completed. It was well tolerated with no serious adverse event. In the US, it has been approved by FDA for phase II clinical trial in patients with multiple sclerosis and asthma. Although no trials have been done in AD patients, results presented in this manuscript highlight a novel plasticity boosting property of RNS60 and suggest that this simple modified saline may be explored for stimulating synaptic plasticity in AD and other dementias.
